# Exploring Agrivoltaics: A Pathway to Climate‐Resilient and Productive Land Use in Northern Bangladesh

**DOI:** 10.1002/pei3.70154

**Published:** 2026-04-27

**Authors:** Shahana Afrose Chowdhury, Nayma Akther Jahan, Md. Sakhawat Hossain Saikat, Haseeb Md. Irfanullah, Samiya Ahmed Selim

**Affiliations:** ^1^ Center for Sustainable Development University of Liberal Arts Bangladesh Dhaka Bangladesh; ^2^ Department of Environmental Studies and Sustainability University of Liberal Arts Bangladesh Dhaka Bangladesh

**Keywords:** agrivoltaics, climate‐smart agriculture, gender‐inclusive agriculture, land‐use efficiency, solar irrigation pumps

## Abstract

The growing demand for food, energy, and water in resource‐constrained regions intensifies land‐use conflicts, where solar photovoltaic (PV) expansion often competes with agriculture. Agrivoltaics, the co‐location of crop cultivation beneath PV systems, offers a potential dual‐use solution to enhance land efficiency. This study presents one of the first agrivoltaic demonstrations in Bangladesh that evaluates the agronomic, economic, and socio‐social feasibility of agrivoltaics through a field‐based comparative experiment conducted at two solar irrigation pump (SIP) sites in Tetulia, Panchagarh district. A controlled plot design was employed in which selected crops were cultivated under PV panels and in adjacent open‐field control plots across two growing seasons (Rabi/winter and Kharif‐I/summer). Crop yields were quantitatively measured and compared, and extrapolation analysis was performed to estimate national‐scale production potential across approximately 45 ha of existing SIP‐covered land. In addition, qualitative data were collected through semi‐structured interviews and focus group discussions (FGDs) to assess farmer perceptions and gender dimensions. Results indicate that seven Rabi crops, including tomato, onion, and garlic, experienced yield reductions of 10%–20% under shaded conditions, whereas shade‐tolerant ginger and turmeric cultivated in Kharif‐I recorded yield increases of 12.3% and 8.7%, respectively. Scaling the pilot findings (0.01 ha) suggests potential seasonal production of nearly 594 t of ginger and turmeric nationwide (45 ha), corresponding to an estimated economic value of approximately US$0.56 million. Qualitative findings revealed strong farmer interest in high‐value crop cultivation under PV panels and indicated enhanced women's participation in crop management, post‐harvest activities, and contributing to household income diversification. The study demonstrates that agrivoltaics can serve as a climate‐smart approach to optimize land use, strengthen food security, and promote renewable energy adoption while creating opportunities for gender‐inclusive agricultural practices in rural Bangladesh.

## 1 Introduction

The world is grappling with the intertwined challenges of food security, energy demand, and sustainable water management. Agriculture consumes nearly 70% of global freshwater withdrawals, while energy systems account for over 70% of greenhouse gas emissions (IEA 2023). Solar photovoltaics (PV) have emerged as one of the fastest‐growing renewable energy technologies, with global installed capacity surpassing 1200 GW by 2023 (IRENA 2023). Yet, the land‐intensive nature of large‐scale solar farms often displaces fertile farmland, intensifying land‐use conflicts, particularly in densely populated and resource‐constrained regions (Curioni et al. 2025). This dilemma calls for integrative solutions that reconcile the competing needs for food production, clean energy, and climate resilience.

Agriculture currently occupies approximately 38% of the global terrestrial surface (Food and Agriculture Organization [FAO] 2022), and further expansion is associated with deforestation, biodiversity loss, and greenhouse gas emissions. At the same time, utility‐scale solar development contributes to what has been described as “energy sprawl,” raising land‐use conflicts between renewable energy infrastructure and food production (Dupraz et al. 2011; Walston et al. 2018). International agencies including FAO and UNEP have therefore emphasized the need for integrated land‐use approaches that minimize trade‐offs between agricultural productivity and renewable energy deployment (FAO 2011; United Nations Environment Programme [UNEP] 2021). In this context, dual‐use systems that enable simultaneous food and energy production are increasingly recognized as critical to sustainable development.

Agrivoltaics (also termed agrovoltaics or Agri‐PV) refers to the deliberate co‐location of agriculture and solar PV systems on the same land, thereby enabling dual production of food and energy. Originating in the 1980s (Goetzberger and Zastrow 1982), agrivoltaics has gained momentum as a response to land scarcity and climate change. PV panels provide partial shading that can reduce crop heat stress, lower evaporation rates, and create favorable microclimates for shade‐tolerant crops (Marrou et al. 2013). Empirical research (Barron‐Gafford et al. 2019) confirms that agrivoltaic shading can reduce soil temperature extremes by several degrees Celsius, improving conditions for root development and nutrient uptake. Such microclimatic buffering is not only beneficial for crop yield stability but also for reducing evapotranspiration, thereby further integrating with irrigation efficiency. Additionally, moderated microclimates can reduce plant stress during peak solar radiation hours, which may help stabilize yields under climate change scenarios characterized by more frequent heat waves. These microclimatic benefits have direct implications for food security, particularly in regions where thermal stress and water scarcity are limiting factors for productivity. By creating a more stable above‐ground environment, agrivoltaics can enhance crop resilience and reduce vulnerability to climatic shocks, which is increasingly important for ensuring dependable food supplies in the context of climate change.

Evidence from Europe, Japan, and the United States shows that agrivoltaic systems can increase overall land productivity by 35%–73% (Dupraz et al. 2011), stabilize yields under climate stress (Di Domenico et al. 2025), and improve soil and water conservation (Barron‐Gafford et al. 2019). Economically, they diversify farmer income by combining crop and electricity revenues, while socially they generate rural employment, empower marginalized groups, and foster community acceptance of renewable technologies (Agir et al. 2023). Furthermore, new research has described how agrivoltaics might enhance irrigation results, not just by providing electricity for pumps but also by changing microclimatic conditions that lower water demand (Chopdar et al. 2025). Improving irrigation efficiency is just as important as increasing the capacity of renewable energy sources in areas with water shortage and climate variability. Agrivoltaics, when integrated with solar irrigation pumps (SIPs), aids in the decarbonization of agricultural water extraction and reduces dependence on diesel‐powered pumping systems (Hossain et al. 2023).

Bangladesh epitomizes the urgency of agrivoltaic adoption. With more than 170 million people living on 147,000 km^2^ and about 50% of the land surface classified as wetlands, the country faces acute land scarcity. According to data from the Bangladesh Bureau of Statistics (BBS), the country lost 1% of its net cropped area. The net cropped area declined from 8.126 million hectares in 2020 to 8.043 million hectares in 2022 (BBS 2023; The Daily Star 2025); the largest drop in a decade (The Daily Star 2025) due to rapid urbanization and industrialization (Kamal and Bin Azam 2024). Agriculture remains vital, contributing 12% to GDP and employing 40% of the workforce (BBS 2024). At the same time, energy demand is expected to double by 2040 (Center for Policy Dialogue [CPD] 2024), straining Bangladesh's fossil‐fuel‐heavy grid.

Bangladesh has made significant investments in SIPs to address this. More than 3400 SIPs will be put in place across the country by 2025, providing close to 60 MWp of clean capacity (Sustainable and Renewable Energy Development Authority (SREDA) 2025). By replacing diesel pumps, these devices cut irrigation costs and greenhouse gas emissions. But thousands of hectares of land beneath PV panels are still mostly unused or underutilized (The Business Standard 2024a, 2024b). Goat rearing, fish farming, and low‐yield crops like corn and lettuce have all been studied under SIP panels in Bangladesh, where pilot projects have shown potential (Shams et al. 2023; Hossain et al. 2023). However, there is limited scientific evidence evaluating crop performance under SIP‐based shading conditions within Bangladesh's agroecological zones (Joy et al. 2023), and few studies integrate agronomic, environmental, and socio‐economic dimensions, including gender participation within agrivoltaic assessments (Shams et al. 2023; Jahan et al. 2024; Bhaduri 2025).

Global evidence underscores the potential of agrivoltaics to enhance food production, renewable energy generation, and rural livelihoods (Widmer et al. 2024). However, crop responses to shading are highly context‐specific, depending on climate, soil type, and cultural practices (Trommsdorff, Dhal, et al. 2022; Trommsdorff, Kang, et al. 2022). Shade tolerance is one of the key factors influencing agrivoltaic success, according to more recent contributions (Giri and Mohanty 2024), which offer detailed evidence on crop‐specific performance across various agroclimatic zones. Similar to this, another study examines yield results for particular crop groups under various irrigation and shade regimes in Odisha, India (Giri and Mohanty 2022). It suggests that modified agrivoltaic designs (such as altering panel spacing and elevation) can reduce yield penalties while maintaining the benefits of energy generation.

In Bangladesh, dominated by rice and sun‐loving vegetables, little is known about which crops can thrive under PV shading. Likewise, while theoretical models (Dinesh and Pearce 2016) suggest strong potential for resource optimization, empirical validation of crop yields, economic viability, and socio‐environmental co‐benefits remains limited. Without such evidence, Bangladesh and countries with solar panel‐based energy transition risk leaving vast tracts of SIP‐covered land underutilized. Despite growing global evidence, empirical research on agrivoltaics in South Asia remains limited, and systematic studies in Bangladesh are particularly scarce (Kamal and Bin Azam 2024; Alves et al. 2026).

Against this background, this study presents one of the first agrivoltaic trials in Bangladesh. The specific research objectives are to:

1. Evaluate crop yield performance under PV panels compared with open control plots across Rabi (winter) and Kharif‐I (summer) seasons.
2. Extrapolate production potential and economic returns from pilot plots to the estimated national SIP land area
3. Assess environmental and socio‐economic benefits, including irrigation efficiency, income diversification, and gender inclusiveness.

By integrating quantitative yield data with qualitative farmer perspectives, this study contributes to the evidence base for agrivoltaics as a resilience‐enhancing strategy for Bangladesh's agricultural and energy sectors. Additionally, this research work makes four main scientific contributions. First of all, it offers one of Bangladesh's first empirical agrivoltaic field trials, producing yield statistics relevant to each season under actual SIP infrastructure. Second, under PV shadowing conditions, it provides a comparative agronomic evaluation across two cropping seasons, differentiating between sun‐dependent and shade‐tolerant crops. Third, it links biophysical performance to livelihood outcomes and gender inclusion by combining qualitative socio‐economic analysis with quantitative yield extrapolation. Lastly, by showing how current solar irrigation infrastructure can be converted into a dual‐use, climate‐smart land management system without the need for further land acquisition, the study improves the food‐energy‐water nexus framework. Through combining agronomic evidence, economic scaling analysis, and socio‐ecological assessment, this research moves beyond technical feasibility to propose agrivoltaics as a scalable development pathway in land‐constrained, climate‐vulnerable contexts.

## 2 Methodology

### 2.1 Study Area

**FIGURE 1:**
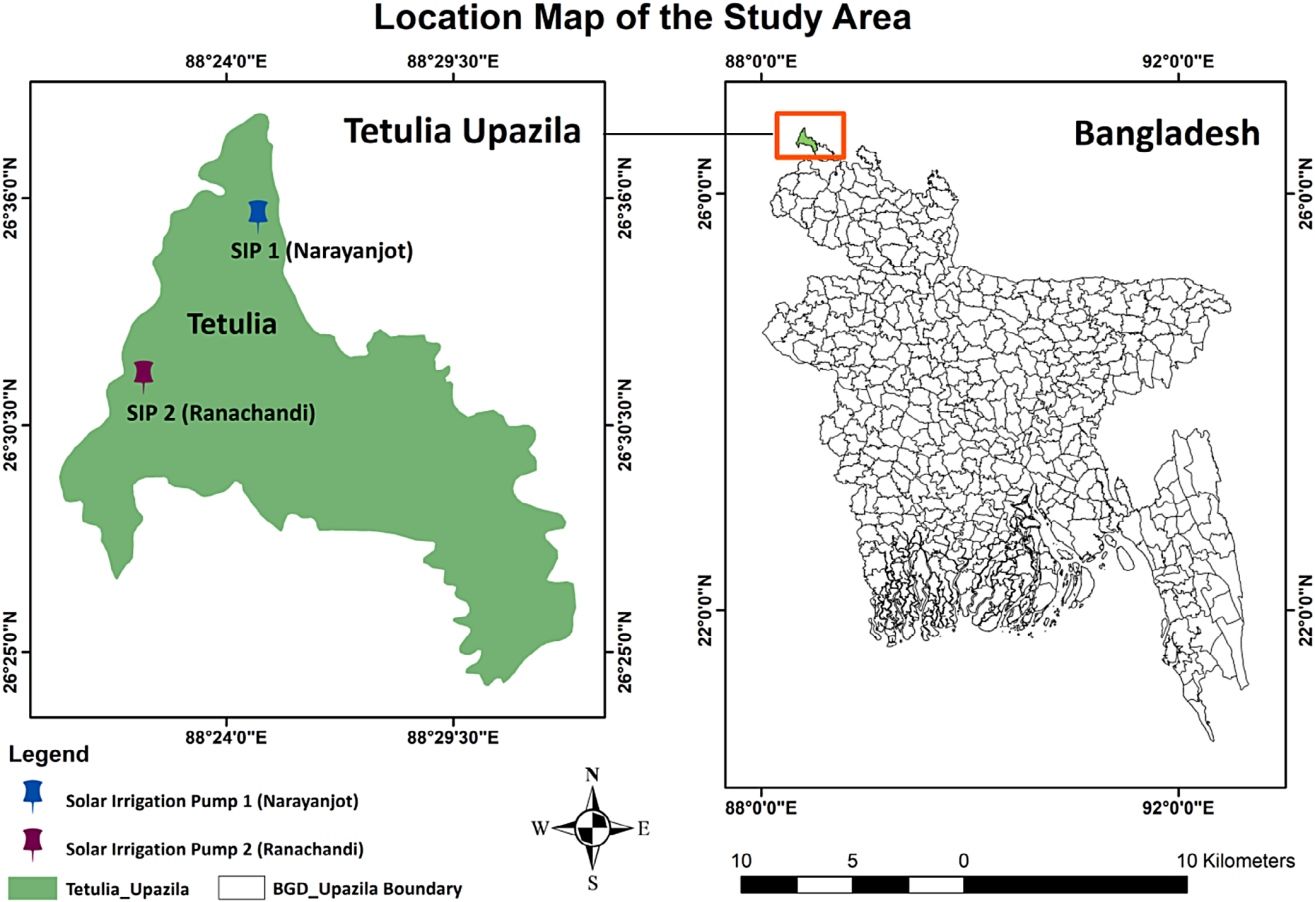
Locations of two solar irrigation pump (SIP) systems in Narayanjot (SIP‐1) and Ranachandi (SIP‐2) villages of Tetulia upazila (subdistrict), Panchagarh.

The study was conducted in Ranachandi and Narayanjot villages in Tetulia upazila (sub‐district) (longitude 26.3333°N, latitude 88.3833°E), Panchagarh district, located in Rangpur division of northern Bangladesh (Figure 1). This region experiences a subtropical climate with distinct Rabi (winter, December–March) and Kharif‐I (summer, March–July) growing seasons. Agriculture dominates local livelihoods, with smallholder farmers cultivating a variety of vegetables, cereals, and spices under both irrigated and rain‐fed systems. Water availability, soil fertility, and access to irrigation critically influence crop productivity in the area. Tetulia was selected as the study site due to its representativeness of northern Bangladesh's farming conditions and the feasibility of integrating solar‐powered irrigation with crop cultivation.

### 2.2 Solar Irrigation Pump (SIP) System

**FIGURE 2:**
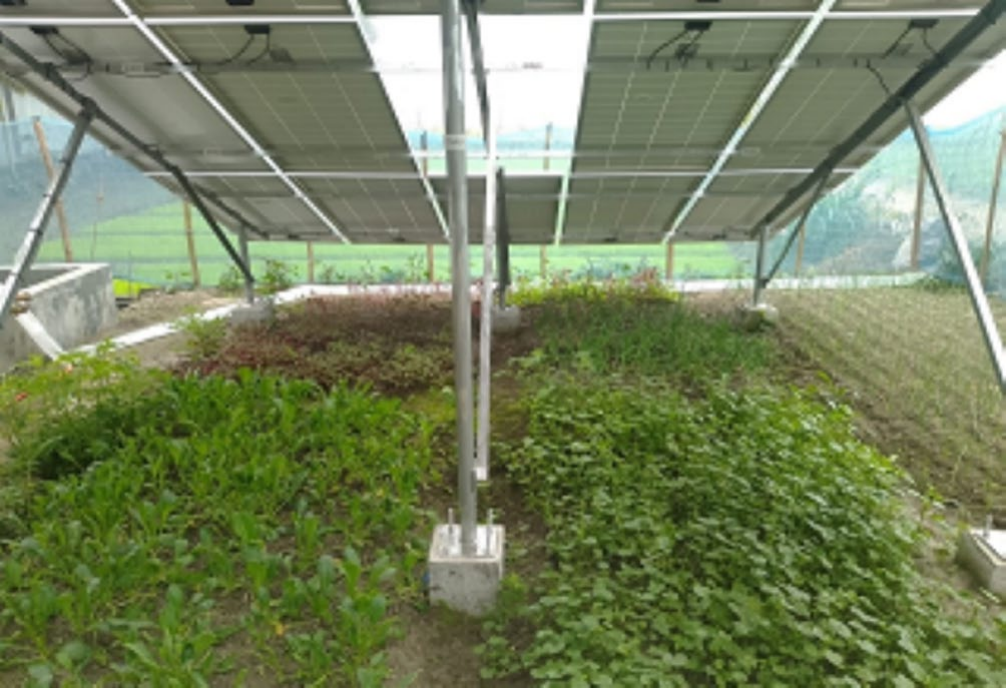
Rabi (winter) crops under the SIP at Tetulia, 28 March 2025.

**FIGURE 3:**
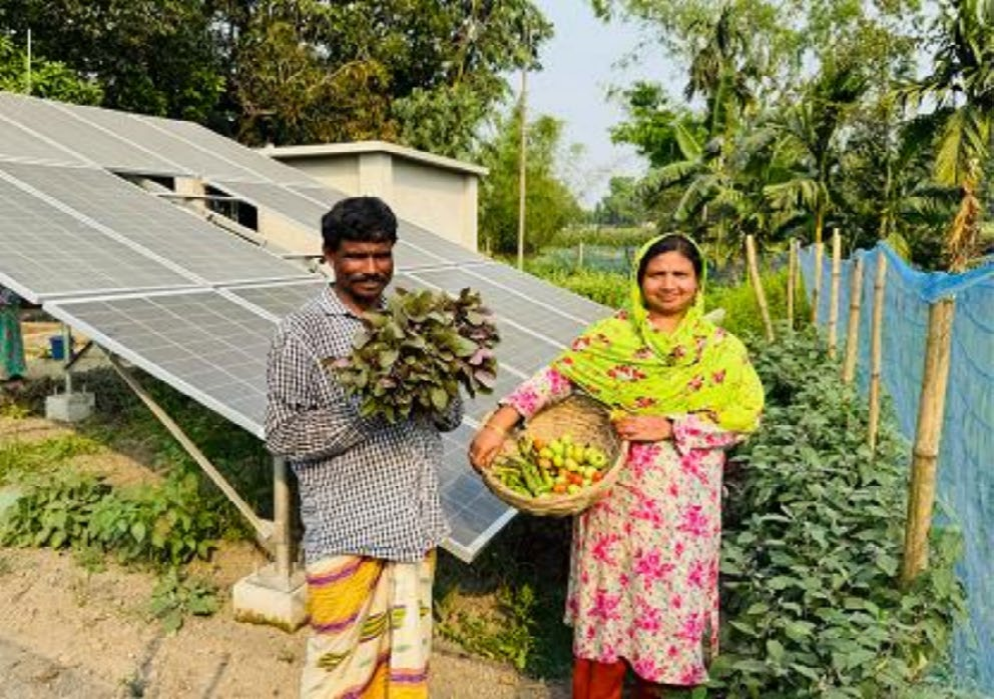
Farmers harvesting crops from agrivoltaics at Tetulia, 28 March 2025.

**FIGURE 4:**
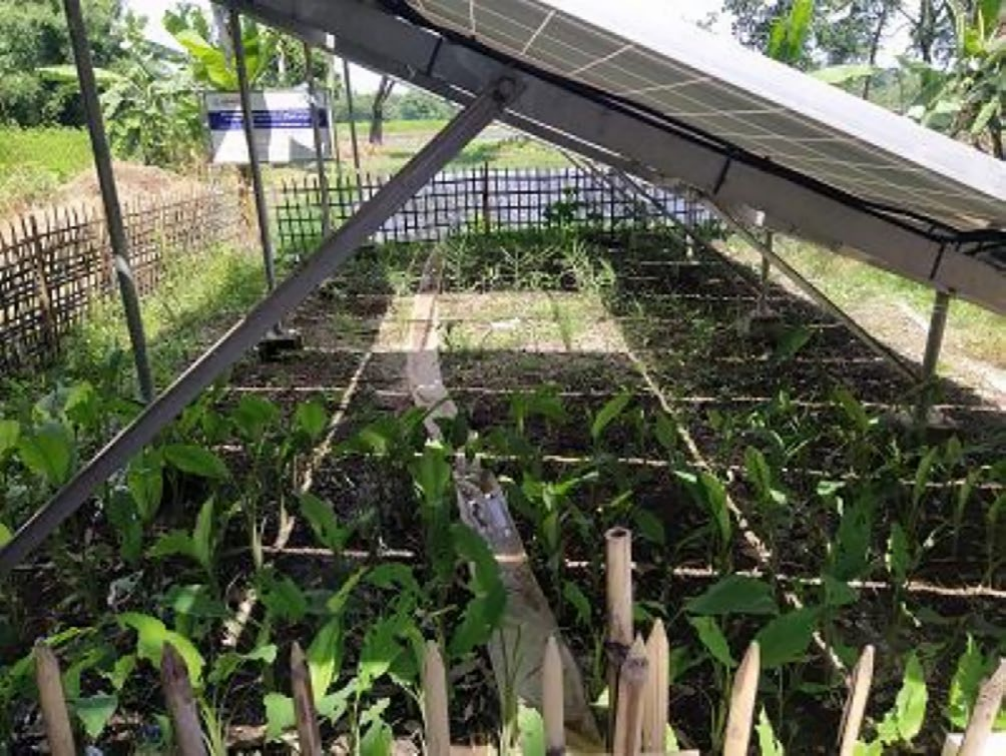
Turmeric and ginger under the SIP at Tetulia, 12 June 2025.

**FIGURE 5:**
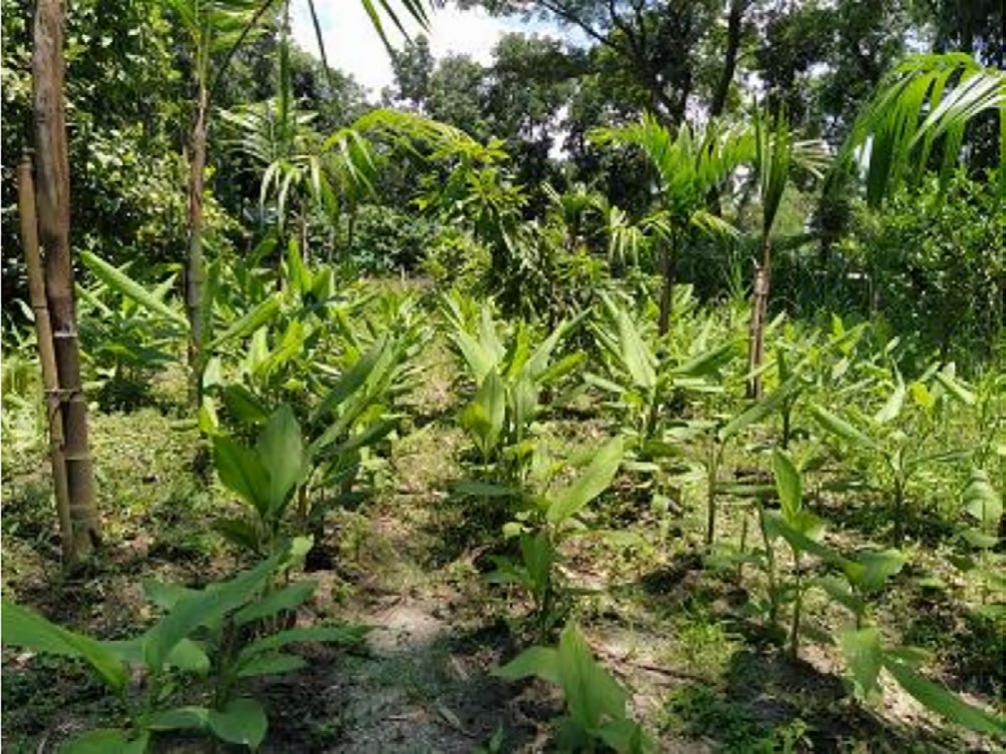
Turmeric and ginger in the control plot at Tetulia, 12 June 2025.

A solar irrigation system (SIS) uses electricity generated from solar photovoltaic (PV) panels to power water pumps for agricultural irrigation. Unlike conventional diesel or grid‐powered pumps, SIS harnesses renewable energy, making it both cost‐effective and environmentally sustainable. The system typically consists of PV panels mounted on a frame, a solar‐powered pump (surface or submersible), and water distribution infrastructure, such as pipes or drip systems (Figures 2, 3, 4, 5). In Bangladesh and many other developing countries, SIS is widely promoted under the SIP program (IWMI 2018). These systems reduce dependency on diesel, lower greenhouse gas emissions, and provide farmers with reliable access to irrigation water, especially in off‐grid rural areas (IDCOL 2023; World Bank 2022). Beyond energy savings, the land beneath PV arrays often remains unused (Santra et al. 2024), creating opportunities for agrivoltaics: the dual use of land for both solar energy generation and crop cultivation.

A research project (April 2024–January 2025), supported by a development partner (former United States Agency for International Development [USAID]), selected two SIP sites, totaling about 0.01 ha of PV panel area. The SIP at Site –1 (Narayanjot) has a 5.5 hp (HP) motor that is driven by a 9.38 kW‐peak (kWp) solar photovoltaic (PV) array. Currently, the system provides domestic water and irrigation to roughly 15 houses, irrigating an area of about 5.35 ha. The 4.69 kWp solar PV system at Site –2 (Ranachandi) supports a 3 HP SIP. This facility enables irrigation over approximately 3.35 ha of agricultural land by supplying water to about 10 houses for both domestic and irrigation uses.

### 2.3 Research Design

The present study employed a comparative experimental design grounded in FAO's Sustainability Assessment of Food and Agriculture Systems (SAFA) framework (FAO 2011), which evaluates sustainability through four key dimensions: (i) environmental integrity, (ii) economic resilience, (iii) social wellbeing, and (iv) good governance. While SAFA provides a broad conceptual roadmap, this research translates it into a practical, field‐based assessment of agrivoltaics in Bangladesh. Environmental integrity was measured by microclimate regulation, enhanced water‐use efficiency, and reduced greenhouse gas emissions through solar‐powered irrigation. Economic resilience focused on crop yields, income potential, and cost savings, with production extrapolated to all fallow land beneath existing solar irrigation systems to highlight the promise of dual land use. Social wellbeing captured locally relevant outcomes, including gender empowerment, improved household nutrition, and farmers' perceptions of agrivoltaic benefits. Good governance was explored through farmer participation, trust‐building, and accountability mechanisms in managing solar‐irrigated plots.

This experiment was designed as an indicative exploration, conducted on a small area (0.01 ha) beneath the solar panels of the two SIP sites, to examine the feasibility of agrivoltaic practices under local conditions. The trials were carried out across two consecutive cropping seasons, the Rabi (winter) and Kharif‐I (summer), with the primary focus on assessing the performance of shade‐tolerant crop varieties. While an array of crops were initially tested, the agrivoltaic assessment was eventually concentrated on ginger ( *Zingiber officinale* ) and turmeric ( *Curcuma longa* ), as both are widely cultivated in the study region and are better adapted to reduced sunlight. This narrowed scope reflects the exploratory nature of the study, aiming to generate preliminary evidence of the compatibility between solar energy generation and agricultural production within smallholder farming systems in Bangladesh.

Two agrivoltaic plots of total 2.5 decimals (0.01 ha) at two SIP sites were compared with adjacent open‐field control plots of the same size (Figure 5), where the same crops were cultivated to compare yield performance. Crops were selected based on seasonal suitability, local relevance, and tolerance to partial shading after consultation with local farmers and the Department of Agriculture Extension Officer of the government. During the Rabi (winter) season (mid‐December 2024–mid‐March 2025), seven commonly cultivated crops were grown in two plots: Chinese mallow ( *Malva verticillata* ), coriander ( *Coriandrum sativum* ), eggplant ( *Solanum melongena* ), garlic ( *Allium sativum* ), onion ( *Allium cepa* ), red spinach (*Amaranthus* spp.), and tomato ( *Solanum lycopersicum* ). In the Kharif‐I (summer) season (March–July 2025), shade‐tolerant crops, such as ginger and turmeric, were cultivated on the same two agrivoltaic plots. The crop selection ensured both practical relevance for local farmers and experimental rigor in assessing the agrivoltaic system's potential.

Alongside the experimental yield comparisons, the study employed a mixed‐methods design that combined interviews and focus group discussions (FGDs) of both male and female farmers. This approach enabled the collection of field‐based agronomic data (crop yield) and basic economic data (market price), while also incorporating farmers' lived experiences, perceptions, and agrivoltaic adoption challenges. Integrating quantitative and qualitative methods in this way is well established in sustainability and farming systems research, as it captures both the technical performance and the social dimensions of an innovation (Creswell and Plano Clark 2018; Menter et al. 2011). In this study, it generated a more holistic understanding of how agrivoltaics contributes to environmental sustainability, economic viability, and social wellbeing in rural Bangladesh. The research design thus provides a robust, evidence‐based assessment of sustainable land use and income generation, directly linked to existing solar irrigation infrastructure.

### 2.4 Data Collection

For crop production assessment, data on yield per hectare and crop quality were recorded over two full growing seasons (Rabi and Kharif‐I) by interviewing the farmers. Using the prevailing local market prices (ginger: Bangladeshi Taka (BDT) 200 per kilogram; turmeric: BDT 100 per kilogram, recorded on 28 March 2025; US$1 = BDT 120), the income potential was calculated per hectare and aggregated for SIP‐covered land area. This provided an estimate of the financial feasibility of combining energy generation with crop cultivation. Observed crop yields were then mathematically extrapolated to the unused fallow land beneath existing solar irrigation installations across Bangladesh, highlighting the potential for additional agricultural output through agrivoltaic integration.

Semi‐structured interviews (*n* = 6) were conducted with smallholder farmers participating in the piloting. Issues covered in the interviews included irrigation efficiency, labor requirements, crop choice, and gender aspects. In addition, two FGDs, each with 8–10 participants (both males and females were present), were organized in both pilot sites to capture collective views on the opportunities and challenges of agrivoltaics. These methods provided insights into farmers' perceptions of adoption, the social acceptability of cultivating beneath solar panels, and potential barriers to scaling.

### 2.5 Data Analysis

Quantitative data on yield were analyzed by calculating percentage differences in yields between agrivoltaic and control plots to evaluate treatment effects. Extrapolated production and revenue estimates were generated by scaling per‐hectare yields to the estimated SIP‐covered land area. There is no directly reported figure for the land area occupied by solar panels of irrigation pumps in Bangladesh. One study notes that 2223 SIPs operating in Bangladesh have an installed capacity of around 45 MWp (Sunny et al. 2023). Therefore, the current SIP installations (approx. 45 MWp) likely occupy around 45 ha of panel area. In Bangladesh, conventional solar PV land‐use estimations can be used in the absence of direct data on panel area for SIPs. Tropical climates typically require 1 ha of capacity per megawatt (MWp) for fixed‐tilt solar arrays (MoPEMR 2018). Assuming a standard installation design (e.g., ground‐mounted, fixed‐tilt), these are approximations. Depending on layout, technology, and efficiency, the actual panel area may be smaller or larger. Nevertheless, in the present study, yields were measured in a total 0.01 ha experimental plots. It was then projected to the estimated 45 ha of SIP‐covered land in Bangladesh. Microsoft Excel was used to do all quantitative analyses.

Qualitative data and information from the interviews and the FGDs were thematically coded after transcription. Codes were grouped into themes, such as “labor shifts,” “income diversification,” “women's empowerment,” and “perceptions of shading effects.” This thematic analysis allowed farmers' narratives to be systematically compared with field observations and quantitative results, ensuring a holistic understanding of agrivoltaic impacts (Braun and Clarke 2006). By combining crop yield records, economic projections, and farmer perspectives, the study produced a comprehensive assessment of agrivoltaics' value addition, resource efficiency, and adoption potential in rural Bangladesh.

It is important to note that this study focused primarily on empirical agronomic performance and socio‐environmental assessment of agrivoltaic systems under operational SIP infrastructure. The analytical framework was designed to compare crop yields under shaded and open‐field conditions, assess seasonal variability, and examine farmer perceptions and livelihood implications. Detailed techno‐economic modeling, such as Land Equivalent Ratio (LER) calculations, net present value estimation, internal rate of return analysis, or payback period modeling, was beyond the defined scope of this research. Consequently, the study emphasizes field‐based yield evaluation and practical feasibility rather than comprehensive financial optimization or system‐level mathematical modeling.

## 3 Results

### 3.1 Comparative Analysis of Crop Yields in 2024–2025

The Rabi (winter) season trials revealed consistent reductions in yields across all seven crops cultivated under the PV panels compared to the open control plots (Table 1). Yield losses ranged from about 11% to 20%, with the sharpest declines observed in bulb‐forming crops, such as garlic and onion, while leafy vegetables (e.g., red spinach) experienced comparatively smaller reductions.

**TABLE 1:** Yield of nine crops in (i) Rabi (winter) and (ii) Kharif‐I (summer) seasons under the solar panels (0.01 ha) and in the control plots (0.01 ha).

Crop	Total yield under the solar panels (in kilogram)	Total yield in the control plots (in kilogram)	Yield difference with the control plots (%)
(i) Rabi season (mid‐December, 2024 to mid‐March, 2025)			
Tomato	9.0	10.5	−14.3
Egg plant	6.3	7.4	−14.7
Lafa shak	5.6	6.8	−17.6
Coriander leaves	1.8	2.1	−14.3
Red spinach	5.0	5.6	−10.7
Garlic	2.5	3.1	−19.4
Onion	5.5	6.9	−20.3
(ii) Kharif‐I season (mid‐March to mid‐July 2025)			
Ginger	82	73	12.3
Turmeric	50	46	8.7

In contrast, the Kharif‐I (summer) trials demonstrated yield advantages for shade‐tolerant crops (Table 1). Both ginger and turmeric produced higher outputs under the PV panels than in open fields. Ginger in particular benefited most, showing more than a 12% increase in yield.

### 3.2 Economic Potential of Agrivoltaics in Bangladesh

In the present study, yields were measured in a total of 0.01 ha experimental plot and then projected to the estimated 45 ha of SIP‐covered land in Bangladesh. The results indicate that ginger could yield approximately 369 t and turmeric about 225 t per season under the total PV panels. At the current market prices, this production translates into revenues exceeding BDT 66.78 million (approx. US$0.56 million) per season (Table 2).

**TABLE 2:** The extrapolated yield, price, and potential revenue for 45 ha (total land area under SIP solar panels countrywide).

Crop	Yield per 45 ha (in kilogram)	Price per kilogram	Total revenue
Ginger	369,000	BDT 120	BDT 44,280,000
		US$1.0	US$369,000
Turmeric	225,000	BDT 100	BDT 22,500,000
		US$0.8	US$187,5000
Total			BDT 66,780,000
			US$556,500

*Note:* The market prices and the currency exchange rate are as of 20 August 2025.

### 3.3 Value Addition and Resource Efficiency

The agrivoltaic pilot conducted on 2.5 decimals of land (~0.01 ha) revealed several benefits extending beyond typical smallholder outcomes. During the qualitative data collection, the farmers emphasized that solar irrigation reduced their dependence on costly diesel or grid‐based pumps, thereby lowering operational expenses and allowing them to retain a larger share of income. This transition not only generated direct economic benefits but also reduced fossil fuel‐related emissions.

The SIP system also improved irrigation practices. With reliable solar‐powered pumping, farmers could avoid both under‐ and over‐irrigation, which supported healthier crop growth. The pilot yielded a total of 34.3 kg of produce across the two sites, demonstrating that productive farming and renewable energy generation can coexist. Onion emerged as the most profitable crop (BDT 265 per kilogram or US$2.2 per kilogram), while garlic (BDT 180 per kilogram or US$1.5 per kilogram) and coriander (BDT 100 per kilogram or US$0.8 per kilogram) achieved the highest per‐kilogram returns. This mix of high‐value and staple crops points to a strategy for enhancing food and income security within limited landholdings.

In addition, farmers observed that the partial shading from panels cooled the soil, reduced evaporation, and shielded plots from heavy rainfall. These micro‐environmental services, though modest at pilot scale, are significant for Bangladesh's climate, where heat and irregular rainfall intensify soil moisture loss. Together, these findings confirm that even on small plots, agrivoltaics can lower costs, conserve resources, sustain yields, and enhance ecosystem resilience, offering clear value for smallholders with scarce land and resources.

### 3.4 Adoption of Agrivoltaics: Farmers' Perceptions

The qualitative analysis, based on six individual interviews and two FGDs conducted in the villages of Ranachandi and Narayanjot, identified five interrelated themes shaping the adoption and socio‐economic impacts of agrivoltaics: (i) awareness and perceptions of the technology, (ii) labor patterns and time use, (iii) income diversification, (iv) women's participation in farm management, and (v) barriers to adoption. The insights from farmers and community members reveal that adoption experiences are deeply influenced by gender roles, household responsibilities, and local socio‐economic conditions, reflecting both the promise and the challenges of integrating agrivoltaics into rural livelihoods.

#### 3.4.1 Awareness and Perceptions

At the outset, both communities had little understanding of what agrivoltaics involved. Respondents and participants initially expressed skepticism, often doubting whether crops could grow beneath the solar panels. A 40 year‐old male respondent from Ranachandi recalled that people thought the project was only about electricity, while younger female participants from Narayanjot admitted that they considered it more of a “risk” than an opportunity. Over time, demonstration plots and peer learning proved crucial in shifting the attitude. A 37 year‐old woman explained how seeing her neighbor's spinach thriving beneath the panels convinced her more than any formal training could. Across both villages, visible success stories helped build trust, encouraging others to experiment with new cultivation practices.

#### 3.4.2 Labor Patterns

The introduction of solar‐powered irrigation significantly altered patterns of labor and time use, especially for women. Several respondents described how the system reduced the need to carry water from distant ponds, freeing time and energy for childcare, household activities, or work in shaded plots beneath the panels. A 22 year‐old woman from Ranachandi emphasized how the solar pump had eliminated hours of physically demanding work. Male respondents also experienced labor shifts, with a 30‐year‐old farmer from Narayanjot noting that they no longer worried about buying diesel or waiting for electricity, as irrigation had become more reliable and predictable. Participants in the focus groups echoed that irrigation scheduling was easier, particularly during the dry season, reducing the stress that typically accompanied water scarcity.

#### 3.4.3 Income Diversification

Economic benefits were evident through opportunities for income diversification. Respondents from both villages explained how leafy greens, coriander, turmeric, and other shade‐tolerant crops were successfully cultivated under the panels alongside rice and other staples. A 38 year‐old man from Ranachandi emphasized that while rice remained central, vegetables grown beneath the panels provided additional income in local markets. Women in Narayanjot shared that these crops not only improved household diets, but also generated small amounts of surplus cash, often used to pay for school supplies or household necessities. This combination of improved nutrition and new income sources reflected the broader livelihood impacts many families hoped the system would sustain.

#### 3.4.4 Women's Participation

Women's involvement in agrivoltaics emerged as a significant yet uneven aspect of adoption. Many women described taking on key responsibilities such as preparing seedlings, selecting crops, and managing vegetable plots beneath the solar panels, while men continued to oversee installation decisions and interact with the SIP engineers. A 23 year‐old woman from Narayanjot explained that she decided which vegetables to plant, though her husband managed the technical components. Discussions in the FGDs indicated that women's participation is gradually becoming more accepted within the community, with participants observing that their opinions now carry greater influence in crop‐related decisions. Nonetheless, gender disparities persist. A 26 year‐old woman shared that she was discouraged from attending a training session by a male relative who considered it inappropriate, underscoring how prevailing social norms still limit women's access to learning opportunities and full participation in agrivoltaic farming.

#### 3.4.5 Barriers to Adoption

Despite enthusiasm for the benefits of agrivoltaics, interviewees and FGD participants identified clear barriers to wider adoption. The high upfront cost of installation was repeatedly emphasized as a major obstacle. For example, one 29 year‐old man from Narayanjot noted that while the SIP system was promising, most farmers could not afford the panels without external support. Technical knowledge gaps also limited confidence, as both men and women reported uncertainty about which crops could thrive under shaded conditions. Women faced additional constraints due to mobility restrictions that prevented them from attending training sessions or technical workshops. As a 27 year‐old female participant in Narayanjot explained, they wanted to learn more, but could not easily travel beyond their villages. These intersecting financial, technical, and cultural barriers highlighted the uneven accessibility of agrivoltaics, raising questions about its long‐term inclusivity and sustainability.

Taken together, the narratives from Ranachandi and Narayanjot villages suggest that agrivoltaics represents more than just a technical innovation; it is a process reshaping everyday farming practices, labor dynamics, and household relations. While the system reduces drudgery, diversifies incomes, and creates new spaces for women's participation, it also reproduces social inequalities and exposes the need for targeted support. Farmers' experiences demonstrate that successful adoption depends not only on the technology itself but also on how it is embedded within local socio‐economic realities.

## 4 Discussion

This pilot study of agrivoltaics in Bangladesh demonstrates the potential of integrating solar irrigation with food production to generate agronomic, economic, environmental, and social value in resource‐constrained settings. While the results indicate substantial opportunities, they also highlight important challenges for scaling, particularly regarding crop choice, system design, and community engagement.

### 4.1 Agronomic, Environmental, and Economic Outcomes

Crop performance under agrivoltaics is primarily influenced by light availability, making crop selection and seasonal alignment critical. The shaded microclimate moderated leaf scorching and improved soil moisture retention, supporting root and tuber development (Raihan et al. 2025). Consistent with international evidence, shade‐intolerant crops, such as cereals, onion, garlic, and tomato, showed yield reductions, whereas turmeric, ginger, leafy vegetables, and other tuber crops performed better under partial shading (Marrou et al. 2013; Barron‐Gafford et al. 2019; Sekiyama and Nagashima 2019). This underscores the importance of crop choice in Bangladesh, particularly during the Rabi season when natural radiation is lower. Adaptive measures, including elevated structures, wider panel spacing, crop sequencing, and intercropping, successfully applied in Japan and Europe (Valle et al. 2017; Weselek et al. 2019), could be localized for Bangladesh. Aligning with the country's NDC 3.0 target of deploying 45,000 SIPs (1000 MWp) by 2035 (MoEFCC 2025), such agronomic optimization can maximize dual food‐energy productivity.

System design is equally important. In humid subtropical conditions, corrosion‐resistant and elevated PV systems are recommended. Hot‐dip galvanized steel or aluminum alloy mounting structures reduce oxidation risks under irrigated conditions (Kalogirou 2014; IRENA 2023). High‐efficiency monocrystalline panels and semi‐transparent or spaced configurations can further balance energy generation with crop light requirements (Marrou et al. 2013).

Successful implementation also requires modified agronomic practices tailored to the agrivoltaic microclimate and crop needs. Raised beds and improved drainage can prevent waterlogging beneath panels by facilitating rapid runoff and reducing soil saturation in shaded zones, while crop spacing and orientation should be adjusted to balance light interception and shading effects on photosynthesis (Abidin et al. 2021; Chopdar et al. 2024). Precision irrigation, particularly drip irrigation, is preferred for managing reduced evapotranspiration and optimizing water use efficiency beneath photovoltaic arrays (Njema et al. 2025). Soil testing‐based nutrient management is critical for maintaining fertility and matching fertilizer applications to crop demand under altered microclimates (Salako et al. 2025). Elevated humidity and microclimatic variability under panels can increase pest and disease risk, so integrated pest management (IPM) practices are essential to sustain crop health (general agronomy consensus on IPM). Finally, structured access pathways between panel rows must be incorporated into system design to protect infrastructure and enable efficient cultivation and harvesting operations without damaging panels or crops (Abidin et al. 2021).

From an environmental perspective, agrivoltaics functions as a multifunctional land‐use system that simultaneously advances climate mitigation, adaptation, and resource efficiency. The partial shading created by PV panels moderates soil and canopy temperatures, reducing evapotranspiration and improving water productivity per unit of crop yield (Elamri et al. 2018; Barron‐Gafford et al. 2019). In contexts characterized by groundwater stress, this reduction in evaporative loss is particularly significant, as it lowers irrigation demand while maintaining crop performance. Observations from the present pilot indicate reduced surface moisture loss and more controlled irrigation through solar‐powered pumping, which enhanced crop resilience during short dry spells. These findings align with international evidence showing that shaded cropping systems can maintain yields with lower water inputs, especially under heat‐stressed conditions (Barron‐Gafford et al. 2019).

Beyond water efficiency, agrivoltaics contributes to climate change mitigation by replacing diesel‐powered irrigation with solar energy, thereby reducing carbon dioxide emissions and particulate pollution. This transition lowers the carbon intensity of agricultural production and improves local air quality. Furthermore, the simultaneous generation of electricity and crops increases total output per hectare, enhancing land‐use efficiency. In land‐scarce and climate‐vulnerable countries, such as Bangladesh, where rainfall variability and soil moisture stress are projected to intensify (Alam et al. 2023), these combined microclimatic and energy benefits position agrivoltaics as a strategic pathway for sustainable intensification without further agricultural expansion or ecosystem degradation.

Economically, agrivoltaics reduce input costs while generating dual revenues (Weselek et al. 2021). Farmers in the pilot reported savings from substituting diesel or grid‐powered irrigation with solar pumping. Although crop revenues were modest given the limited pilot scale (34.3 kg total output from 0.01 ha), the highest margins came from onions, garlic, and coriander. More importantly, shade‐tolerant high‐value crops, such as turmeric and ginger, showed promise for significantly improving household margins when extrapolated to larger scales. For smallholder farmers, such returns are transformative, as they represent multiple years of typical farm income condensed into a single‐cropping season.

The national implications are particularly compelling for spice production. Bangladesh consumes around 5.85 million tonnes of spices annually but produces only 4.93 million tonnes, leaving a 0.92 million‐tonne shortfall filled through imports (The Daily Star 2023a). Bangladesh Bank data show that in the 2022–2023 fiscal year, the country spent US$417.30 million on spice imports, while exporting only US$42.38 million (The Daily Star 2023b). If agrivoltaic systems were scaled to boost spice cultivation, particularly turmeric and ginger, production could rise from 4.93 to 5.50 million tonnes in five years, reducing imports by 0.57 million tonnes and saving approximately US$570 million annually. Beyond stabilizing domestic prices, this could create export opportunities, particularly through value‐added processing and packaging. The Bangladesh Agricultural Research Institute (BARI) already includes a Spices Research Program within its crop research portfolio, and its Annual Research Report 2022–2023 documents ongoing trials with onion, garlic, chili, and other spices (BARI 2023). Additionally, a study on the financial and economic profitability of selected spice crops reported that national production rose from 0.418 million tonnes in 2001–2002 to ≈2.996 million tonnes in 2019–2020, illustrating long‐term growth and the potential for scaling (Rashid et al. 2022). Agrivoltaic interventions could therefore complement ongoing research and development (R&D) by integrating crop productivity with renewable energy for irrigation, drying, and processing.

### 4.2 Socio‐Economic Impacts, Adoption Pathways, and Study Limitations

The present pilot also emphasized agrivoltaics' social aspects. Households decreased economic risk and diversified their sources of income by integrating solar irrigation with crop production. Women actively engaged in farm management and irrigation, extending their decision‐making responsibilities in ways that defy gender stereotypes. This is consistent with research showing that women can be empowered and labor patterns can be changed by having access to irrigation and renewable technology, but only if these technologies are created with inclusivity in mind (Parveen and Leonhäuser 2004; Closas and Rap 2017). For instance, depending on governance structures, solar irrigation initiatives in India have produced varying gender outcomes (Baruah 2017). According to the present Bangladesh pilot, agrivoltaics may provide equitable benefits through deliberate inclusion initiatives. The participatory adoption process observed in this study suggests that agrivoltaics can foster community ownership when farmers witness tangible benefits. Importantly, the system reduces fuel dependency, protecting farmers from volatility in global energy markets. The technology is significant because it lessens reliance on fuel, shielding farmers from fluctuations in international energy contracts. As a result, agrivoltaics is a socio‐technical innovation that is integrated into rural development systems rather than just a technical intervention.

Acceptance in the community was another important factor. After farmers saw measurable outcomes, agrivoltaics, which had been initially viewed with suspicion, acquired support. This supports Rogers' (2003) Diffusion of Innovations theory, which highlights the importance of trialability and observability in adoption. According to Feder and Umali (1993), demonstration plots that lower uncertainty are well received by rural populations, who are frequently risk averse and dependent on peer networks. By integrating new technology into established social structures, lead farmer models, farmer field schools, and participatory trials improve adoption, according to data from South Asia and sub‐Saharan Africa (Anderson and Feder 2007; Spielman et al. 2011). Adoption has also been demonstrated to be sustained over time through participatory governance, in which farmers actively participate in planning and management (Hellin 2012). Therefore, the pilot highlights the value of group participation and social learning in expanding agrivoltaics in rural Bangladesh.

This study also carries several limitations. The findings are based on only two SIP sites with small areas under solar panels, making results context‐specific and exploratory, rather than generalizable. Data were derived primarily from field observations and farmer assessments, crop yield and local market values of the crops, which carry subjective elements. The pilot was also limited to northern Bangladesh, where agro‐ecological conditions differ from other regions. While these constraints limit the scope of inference, they underscore the need for broader, multi‐site studies covering diverse cropping systems, panel designs, and governance models.

Despite these limitations, the exploratory findings provide a strong case for expanding agrivoltaics as a pathway to climate‐resilient, energy‐integrated, and socially inclusive farming systems. Scaling efforts should focus on adaptive agronomic design, high‐value shade‐tolerant crops, gender‐sensitive governance, and participatory adoption models to maximize both livelihood and national economic benefits. Thus, the observed growth in community acceptance of agrivoltaics through demonstration‐based adoption is consistent with the broader evidence on rural innovation diffusion: technologies are more likely to scale when farmers learn collectively, see practical benefits firsthand, and engage in participatory governance processes that build trust and reduce adoption risks.

### 4.3 Policy and Scaling Implications for Agrivoltaics

Scaling agrivoltaics in Bangladesh requires integrated policy support. Globally, progress has been strongest where energy and agricultural policies are aligned (Dupraz et al. 2011; Weselek et al. 2019). In Bangladesh, however, energy and agriculture remain siloed. A water‐energy‐food (WEF) nexus framework could help synchronize investments, ensuring that renewable energy expansion also delivers food security and rural livelihoods. Three specific policy priorities emerge from this study as described below:

#### 4.3.1 Strengthening Agricultural Extension Services

Extension services play a pivotal role in helping farmers navigate new production environments under PV panels, including crop suitability, irrigation scheduling, and adaptive agronomic practices. Evidence from agricultural extension in Asia and Africa shows that well‐designed, locally adapted extension increases adoption of sustainable technologies by reducing information asymmetries and tailoring recommendations to farmer needs (Anderson and Feder 2007). Farmer field schools and participatory extension approaches, in particular, have been effective in enhancing adoption of climate‐smart practices by fostering experiential learning and peer‐to‐peer exchange (Davis et al. 2012). Integrating agrivoltaics into extension curricula will therefore be critical for ensuring farmers can make informed decisions about crop choices, water management, and technology adaptation.

#### 4.3.2 Financing Mechanisms to Expedite Agrivoltaics Adoption

The high upfront costs of agrivoltaic infrastructure represent a key barrier to smallholder participation. Experiences from renewable energy and irrigation technologies demonstrate that concessional loans, subsidies, and public‐private partnerships can substantially reduce adoption risks and expand access (Burney et al. 2010). For example, subsidy programs in India for SIPs significantly improved farmer uptake while reducing dependence on diesel‐based systems (Shah et al. 2017). Similarly, microcredit and cooperative financing models have helped marginalized farmers access inputs and technologies otherwise beyond their means (Khandker and Koolwal 2016). Designing blended‐finance packages that combine concessional loans with targeted subsidies could accelerate agrivoltaics adoption while maintaining fiscal sustainability. Investment in research and development (R&D) is also needed to identify optimal panel configurations and crop mixes for Bangladesh's diverse agroecological zones. Integrating this research into renewable energy programs would help adapt agrivoltaics to local contexts while maximizing productivity.

#### 4.3.3 Gender‐Sensitive Agriculture and Energy Policies

Policies must also recognize and address gendered constraints in access to land, training, and resources. Women are frequently excluded from agricultural innovation processes due to structural barriers in extension, credit, and decision‐making (Quisumbing et al. 2014). Evidence shows that gender‐sensitive extension services and training programs not only increase women's participation but also improve household food security and technology adoption rates (Meinzen‐Dick et al. 2014). In renewable energy projects, gender‐responsive approaches have been linked to more equitable benefit‐sharing and greater long‐term sustainability (Clancy et al. 2012). Thus, agrivoltaic policies should explicitly ensure women's involvement in training, access to finance, and governance structures to maximize both equity and system‐wide benefits. Apart from policy, agrivoltaic systems naturally provide gender‐responsive potential by establishing diverse livelihood zones that allow women to manage crops, run nurseries, and process value‐added products all on the same piece of land. The shade‐tolerant cropping zones beneath panels often allow flexible, less labor‐intensive agricultural tasks that align with women's existing household and caregiving roles, thereby enabling their greater participation in productive work.

### 4.4 Agrivoltaic and the Sustainable Development Goals

Agrivoltaics aligns strongly with multiple Sustainable Development Goals (UN SDGs 2015). It contributes to SDG 2 (Zero Hunger) by enhancing agricultural productivity and supporting high‐value crop cultivation. Through solar‐powered irrigation, it advances SDG 7 (Affordable and Clean Energy) by expanding renewable energy access in rural areas. Reduced fossil fuel use and enhanced climate resilience support SDG 13 (Climate Action). Improved water‐use efficiency under SIP systems aligns with SDG 6 (Clean Water and Sanitation). Furthermore, increased participation of women in irrigation management and crop value chains directly advances SDG 5 (Gender Equality). By increasing land‐use efficiency and promoting sustainable production systems, agrivoltaics also contributes to SDG 12 (Responsible Consumption and Production). The multi‐SDG alignment underscores its value as an integrated development intervention rather than a single‐sector solution.

## 5 Conclusion

This study presents one of the first empirical evaluations of agrivoltaics under operational SIP infrastructure in Bangladesh. The findings demonstrate that crop response under PV shading is species‐specific: shade‐intolerant crops experienced moderate yield reductions, while shade‐tolerant crops such as ginger and turmeric showed stable or improved performance. These results confirm that appropriate crop selection and seasonal alignment are critical to agrivoltaic viability, particularly under Rabi season light constraints. When extrapolated to the national SIP expansion target, agrivoltaics present measurable potential to enhance dual land productivity without additional land conversion.

Beyond agronomic outcomes, the system offers environmental and socio‐economic advantages. Partial shading improved soil moisture retention and water‐use efficiency, while solar‐powered irrigation reduced dependence on diesel, lowering carbon emissions and production costs. The co‐generation of food and renewable energy strengthens land‐use efficiency in land‐scarce contexts and contributes to livelihood diversification, including enhanced participation of women in agricultural management.

While this study establishes empirical feasibility and practical relevance, future research should incorporate integrated techno‐economic and land‐use efficiency modeling to deepen quantitative evaluation. Specifically, calculation of Land Equivalent Ratio (LER) would provide a more precise assessment of combined food and energy productivity per unit area. Revenue optimization modeling, life‐cycle cost analysis, and payback period estimation under different panel configurations and crop combinations would further clarify long‐term financial viability. Expanded multi‐site trials across agro‐ecological zones would strengthen policy guidance for scaling agrivoltaics under Bangladesh's national solar irrigation expansion strategy.

Overall, the study demonstrates that agrivoltaics represents a viable climate‐smart pathway for enhancing land productivity, strengthening rural resilience, and advancing sustainable energy‐agriculture integration in Bangladesh.

## Funding

The authors have nothing to report.

## Conflicts of Interest

The authors declare no conflicts of interest.

## Supporting information


**Data S1:** pei370154‐sup‐0001‐Supinfo.xlsx.

## Data Availability

The data generated and analyzed during this study that are presented in the manuscript are available in the Supplementary_Data_Agrivoltaics_Bangladesh.xlsx file.
